# Emoji for Food and Beverage Research: Pleasure, Arousal and Dominance Meanings and Appropriateness for Use

**DOI:** 10.3390/foods10112880

**Published:** 2021-11-22

**Authors:** Sara R. Jaeger, David Jin, Grace S. Ryan, Joachim J. Schouteten

**Affiliations:** 1 The New Zealand Institute for Plant and Food Research Limited, Mt Albert Research Centre, Private Bag 92169, Auckland 1142, New Zealand; sara.jaeger@plantandfood.co.nz (S.R.J.); david.jin@plantandfood.co.nz (D.J.); grace.ryan@plantandfood.co.nz (G.S.R.); 2 Department of Agricultural Economics, Ghent University, Coupure Links 653, 9000 Gent, Belgium

**Keywords:** emoji, emotions, pleasure, arousal, dominance, PAD model, consumer research

## Abstract

Emoji have been argued to have considerable potential for emotion research but are struggling with uptake in part because knowledge about their meaning is lacking. The present research included 24 emoji (14 facial, 10 non-facial) which were characterized using the PAD model (Pleasure-Arousal-Dominance) of human affect by 165 consumers in New Zealand and 861 consumers in the UK. The results from the two countries were remarkably similar and contributed further evidence that emoji are suitable for cross-cultural research. While significant differences between the emoji were established for each of the PAD dimensions, the mean scores differed most on the Pleasure dimension (positive to negative), then on the Arousal dimension (activated to deactivated), and lastly on the Dominance dimension (dominance to submissive). The research also directly measured the perceived appropriateness of the 24 emoji for use with foods and beverages. The emoji face savoring food, clapping hands and party popper were in the top-5 for the highest appropriateness in food and beverage context for both studies, as was a strong negative expression linked to rejection (Study 1: face vomiting; Study 2: nauseated face). On the other hand, zzz and oncoming fist were considered as the least appropriate to be used in a food and beverage context in both studies. Again, the results from the UK and NZ were in good agreement and identified similar groups of emoji as most and least suitable for food-related consumer research.

## 1. Introduction

### 1.1. Emoji in Food-Related Consumer Research

As a means to gain additional insights about consumers’ product experiences, measurement of emotional associations to foods and beverages (F&B) has increased in the past decade [1,2]. Questionnaires are primarily used, and regarded as the default measurement approach [3]. They can be implemented using emotion words or emoji, and we focus here on the latter which are less studied than emotion words [4] but have high relevance in consumer research as graphical representations that convey emotions and feelings through their resemblance with real objects [5]. 

Emoji are now widely used on social platforms and in digital communications have become well known by many in the general public [6]. This has the advantage that participants in consumer research are likely to be used to expressing emotions and feelings through emoji which, relative to emotion words, positively contributes to ecological validity [4,7]. As emoji are non-verbal, they enable consumers to express emotions that otherwise cannot be expressed with words or that would be expressed differently [8]. The potential of emoji in consumer research has been illustrated by several studies showing that they can discriminate between F&B products (e.g., [9,10,11]) and increase food choice prediction [12]. However, it has been pointed out that knowledge is lacking about which emoji are appropriate to be used in the context of F&Bs and eating/drinking consumption situations more broadly [4,13]. This fits with the notion that textual context could reduce ambiguity in interpretation of some emoji [14]. Therefore, there is a need to establish suitability for individual emoji in food-related consumer research.

### 1.2. Dimensional Meanings of Emoji: Pleasure, Arousal and Dominance

Despite empirical evidence pointing to good similarity between the meanings and interpretations that research participants associate to different emoji [15,16], there is nonetheless limited knowledge on emoji meanings in the context of food-related research, and this has been suggested as a major barrier for uptake [4,13]. Furthermore, one needs to consider that emoji meanings can be established by obtaining more information about the verbal meaning of an emoji (e.g., by a CATA question or assessment of the use of the emoji in a certain food context) but also by assessing the meanings of the emoji related to the main dimensions of emotions. To bring a new aspect to this pursuit, we focus in this paper on emoji meaning on such underpinning dimensions of emotions and human affect [17] rather than profiling by individual emotion words.

Dimensions of emotions have not been an area of focus in food-related consumer research [18] and emphasis has de facto been on the valence dimension (positive to negative), where even emotion words with negative valence were underrepresented in early works (e.g., [19]). In more recent times, the arousal dimension (activated to deactivated) has been given greater attention, for example by the development of a circumplex-inspired emotion word questionnaire that purposefully spans the valence × arousal space [20]. Further, Self-Assessment Manikins (SAM: [21]) have been used to measure establish meanings for the dimensions of valance and arousal in the USA [22] and China [23].

However, the dominance dimension (dominance to submissive) remains largely neglected still. This dimension can be seen as a “power” dimension which is related to the extent that a person is in control of a situation and the people in that situation [24]. The dominance dimension can distinguish between similar arousal levels. For example, fear and anger are both high in arousal but fear is low in dominance while anger is high in dominance [8]. Data from a projective mapping exercise using emoji with pre-adolescents suggest that the dominance dimension might also be important for the meaning attached to emoji [8]. The authors hypothesized that, compared to adults, higher relevance of the dominance dimension among pre-adolescents might be due to a focus on situational control (thus dominance) rather than psychological activation.

When considering the different dimensions of human affect, the PAD model (Pleasure, Arousal and Dominance: [25]) has relevance since it incorporates the three aforementioned dimensions and has been widely used to examine human affect and emotion [17]. The PAD model has been validated as appropriate for studying consumer emotions and consumer behavior in different consumption contexts, including food-related settings [26,27], and is therefore appropriate for research into emoji meaning.

### 1.3. Research Aims and Contributions

Building on the research summarized above, and the recent direct recommendations of Jaeger, Vidal and Ares [4] and Schouteten and Meiselman [13], the present research had two objectives: (1) contribute further knowledge regarding emoji meanings, and (2) establish the extent to which consumers find certain emoji suitable in the F&B context.

We extend previous research by using the PAD scale to measure three dimensions of meaning and by including some different emoji to those previously considered. We also obtain data in a country where emoji meanings regarding the main emotional dimensions (valence, arousal, and power) have not previously been established: New Zealand (NZ) and the United Kingdom (UK).

Finally, we directly measure the perceived appropriateness of individual emoji in the context of F&Bs and associated consumption occasions. It should be noted that the goal was to explore the application of the PAD meanings of the selected emoji in two countries, rather than directly comparing the PAD meanings in these two countries.

## 2. Materials and Methods

### 2.1. Participants

Study 1 was conducted in NZ at a central location testing facility in Auckland. Adult participants (*n* = 180) (50% female, 20–65 years old) were recruited by a professional company. Study 2 took place in the UK (England, Scotland, Wales, and Northern Ireland) with participants (*n* = 1047) (50% female, 18–65 years old) who had self-registered with an ISO-accredited web panel provider. The Supplementary Material has full participant details.

Both studies were covered by a general approval for sensory and consumer research from the Human Ethics Committee at the New Zealand Institute for Plant and Food Research (PFR). Participants were assured that their responses would remain confidential. As compensation, participants in Study 1 received cash while those in Study 2 earned reward points and promotional offers.

### 2.2. Brief Overview of Empirical Approach

There were three parts to the empirical approach that participants took part in. The first was to complete the 18 PAD scales (i.e., emoji meaning) for several emoji. Then participants completed questions relating to Objective 2. These captured emoji specific use and suitability for F&B occasions. Finally, there were questions about emoji use and attitudes, as well as demographic and socio-economic questions. The procedures are described in full below, starting with the selection of emoji to be included in the research.

### 2.3. Emoji Selection and Evaluation

#### 2.3.1. Emoji

A candidate set of emoji were proposed by two authors and revised through discussion with other authors and colleagues. The number of emoji included in the research (*n* = 24) was determined by constraints of Study 1 and the target was to have each emoji evaluated by ~40 participants.

**Figure 1:**
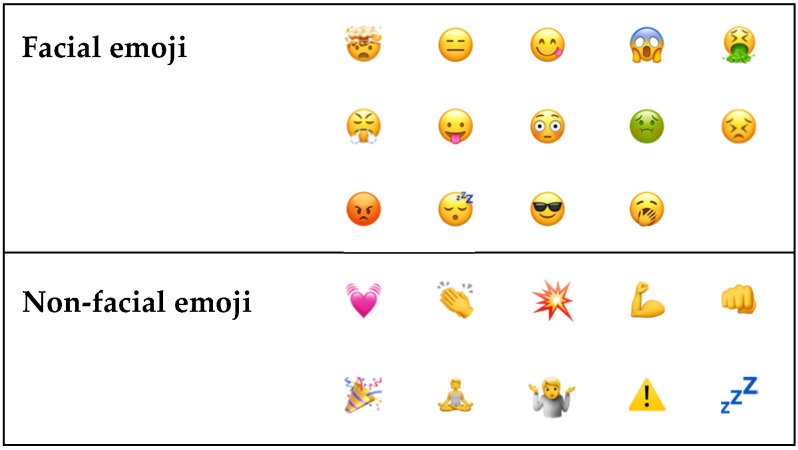
Emoji included in the research.

The same set of 24 emoji was used in both studies (Figure 1). Their selection process was guided by several criteria, foremost seeking to span each of the PAD dimensions, through a combination of emoji previously used in food-related research [8,22], those known to be popular on social media in situations relating to eating and drinking [28] and emoji not previously considered in this research domain (e.g., 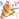, 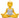, 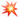). Facial emoji were used primarily, but where a greater range in one or more of the PAD dimensions were expected by the inclusion of non-facial emoji, these were selected (42%). Inclusion of non-facial emotion was warranted since some of the most-used emoji on Twitter are non-facial (https://emojitracker.com/, (accessed on 1 April 2021)), and some have obvious links to emotional affect (e.g., red heart (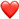), thumbs up sign (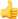) and thumbs down sign (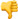)) [29]. Moreover, some previous studies excluded non-facial emoji [22], so less is known about their meaning.

#### 2.3.2. PAD Scale

The PAD scale from Mehrabian and Russell [25], which is a well-established and valid measurement tool valid for the scientific study of human emotions [17], was used to measure emoji meaning (Obj. 1).

Specifically, each emoji was evaluated on the 18 semantic differentials (Table 1), which comprised six items for each of the three dimensions P (Pleasure), A (Arousal), and D (Dominance) (Obj. 1). These were implemented accordingly using 9-pt scales. There was one minor change: for P6, based on Detandt, Leys [30], the right anchor (9) was retained as ‘bored’ while the left anchor (1) was changed from ‘relaxed’ to ‘amused’.

In keeping with Mehrabian and Russell [25], *Pleasure* was regarded as a feeling along a continuum ranging from unhappiness to extreme happiness (i.e., valence). The *Arousal* dimension was interpreted as a mental activity that can be described along a single dimension ranging from sleep to excitement. Lastly, *Dominance* was depicted as being related to feelings of control along a continuum from submissive to dominance.

#### 2.3.3. Emoji Specific Use and Suitability for F&B Occasions

In addition to consumers’ evaluation of the emoji on the PAD scales, the emoji were also assessed in relation to use characteristics specific to F&Bs and/or eating/drinking situations (Obj. 2). Three questions were used, where the first asked participants to indicate frequency of use of the focal emoji when sending messages, emails, etc. A 5-pt response scale was used with anchors: 5 = ‘always or almost every time (>90%)’, 4 = ‘frequently (more often than not, 65–90%)’, 3 = ‘sometimes (not all the time, but neither rarely, 35–65%)’, 2 = ‘infrequently (generally not, but on occasion, 10–35%)’ and ‘1 = never or very infrequently (<10%)’. This question was only included in study 1. Next, participants were asked to indicate whether or not they could imagine a food/beverage or eating situation where using this emoji would be appropriate. The final question pertained to past use and participants responded yes, no, not sure, to the question of whether they recalled having used the focal emoji for a food, beverage or eating situation.

#### 2.3.4. General Emoji Use and Attitudes

General emoji use was measured with the question: “In general, how often do you use emoji when sending messages, emails etc.?” which had the same five answer categories as used above for the emoji-specific use question.

A set of eight statements capturing general attitudes to emoji were scaled on fully labeled 7-pt Likert statements with endpoint anchors 1 = ‘disagree strongly’ and 7 = ‘agree strongly’. The statements were: ‘Emoji are more appropriate in private than professional communications’, ‘I generally use a small set of emoji over and over again’, ‘Using emoji helps me to express my moods/emotions’, ‘My favorite emoji are “face emoji” (e.g., 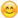, 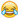, 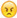)’, ‘I like to use many different emoji, of all kinds’, ‘I consider myself to be emoji savvy and literate’, ‘Emoji are fun to use and receive’, and ‘In computer-mediated communications, emoji help me to better express myself’.

### 2.4. Data Collection Procedures

Emoji were presented sequentially in both studies, following a Williams design (Study 1) or in randomised order (Study 2). Pen and paper ballots were used in Study 1, with the emoji shown at the top of the page in colour (4.2 × 4.2 cm) and the 18 PAD scales below. These were followed by the questions pertaining to emoji specific uses and perceptions. Each participant evaluated six emoji, and once completed a short ballot followed comprising questions relating to the general use of emoji. The procedure was very similar in Study 2, with the exception that each participant evaluated three emoji and each of the 18 PAD scales were presented horizontally and shown individually (Part 2 of Supplementary Material). Part 3 of Supplementary Material has details on the allocation of emoji to participants. The presentation order of PAD scales varied across respondents, in accordance with Ares, Reis et al. (2015) in Study 1 and randomly in Study 2.

Study 1 took part in a central location facility and participants were seated in standard sensory testing booths. Pen and paper ballots were used in Study 1 as the CLT location does not have access to computerized data collection infrastructure. Participants in Study 2 completed the survey in a location of their choosing using a desktop/laptop computer or tablet. In both studies, the data were obtained as part of surveys/research sessions that included tasks other than those described here (not considered further due to lack of relevance). Demographic and socio-economic information was obtained as the final part of the survey/session. Participants typically took 10–15 min to complete the study.

### 2.5. Data Analysis

The data for each study were analysed separately, and all analyses were performed in XLSTAT v.2020.5.1 [31] using a 5% significance level.

The first step was to exclude participants who stated that they never used emoji or only did so infrequently (i.e., <10% of communications). It was regarded as non-sensical to include such participants to address the aims of the research. The effective sample sizes were: Study (1) *n* = 165, with each emoji being evaluated by 40 to 43 participants, and Study (2) *n* = 861 with each emoji being evaluated by 99 to 116 participants.

Objective 1: The 18 PAD variables (Study 1 and Study 2) were input to a one-way analysis of variance (ANOVA) using emoji as a fixed factor and participants as a random factor. Tukey’s HSD was used for post hoc tests. Principal components analysis (PCA) was performed using the correlation matrix based on means as input. RV coefficients were calculated to determine the similarity between the PCA matrixes (3 dim.) of the emoji and PAD variables for the NZ and UK study. One-way ANOVA was performed for each of the three dimensions (P, A and D) and supplemented with post hoc tests as above, after confirming that Cronbach alpha values exceeded the typical 0.7 threshold (α for, respectively, Pleasure, arousal and dominance was 0.95, 0.90 and 0.79, in Study 1 and 0.93, 0.88 and 0.76 in Study 2).

Objective 2: Emoji use responses (Study 1) were also analysed using a one-way ANOVA, while citation frequencies were calculated for responses relating to eating and drinking. Correlation analyses were used to determine the association between emoji-specific use frequencies and perceived appropriateness for use in situations relating to F&Bs or eating and drinking.

## 3. Results

The results for Study 1 and Study 2 were very similar and are jointly presented, progressing from Objective 1 (dimensional emoji meanings) to Objective 2 (emoji appropriateness and use in the context of F&B consumption).

### 3.1. Emoji Characterisation on PAD Variables

**Figure 2:**
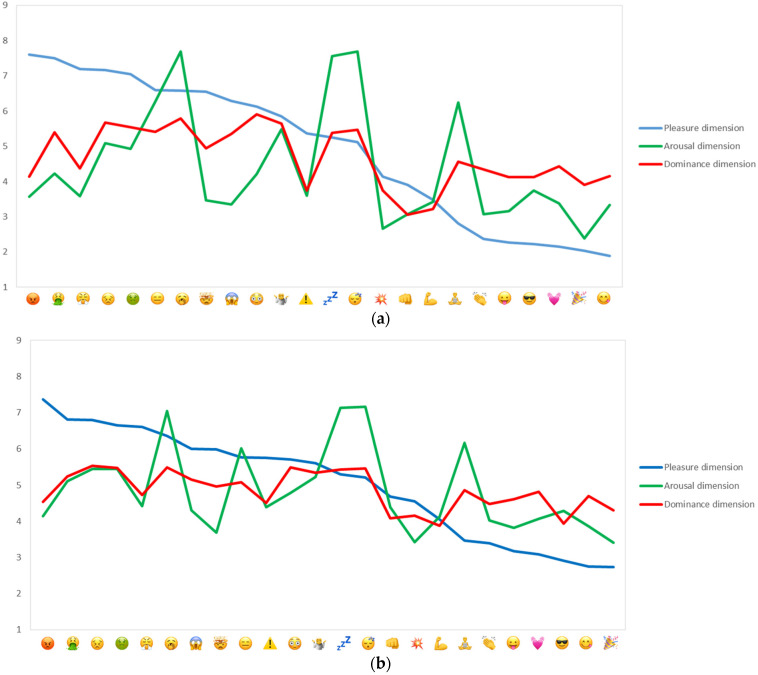
Emoji characterization by PAD dimensions for: (**a**) Study 1 (NZ) and (**b**) Study 2 (UK). *Pleasure*—low anchor (1) is associated with pleasure and high anchor (9) is associated with displeasure; *Arousal*—low anchor (1) is associated with arousal and high anchor (9) is associated with nonarousal (A); *Dominance*—low anchor (1) is associated with dominance and high anchor (9) is associated with submissiveness. Means are sorted by value for *Pleasure* within studies.

For the main insights relating to Objective 1, the 18 PAD variables were averaged to their three respective dimensions. Significant differences (*p* < 0.001) between the 24 emoji were established (Parts 4 to 6 of Supplementary Material contains detailed results by study incl. Tukey HSD post hoc tests). Figure 2 showed that the span in mean scores across the 24 emoji was larger in Study 1 than Study 2, although it should be noted that the same patterns were observed in both studies. For example, the top-5 ranked emoji for pleasure and displeasure were identical (respectively, party popper (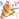), face savouring food (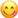), smiling face with sunglasses (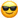), face with tongue (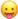) and pouting face (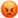), face vomiting (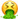), persevering face (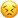), nauseated face (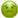), face with steam from nose (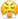). For the deactivated pole of the *Arousal* dimension, the top-5 ranked emoji were also identical (yawning face (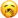), sleeping face (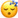), zzz (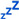), person in lotus position (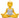) and expressionless face (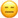)), as were three of the top-5 ranked emoji for the activated pole of *Arousal* (party popper (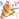), collision (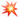), face with tongue (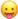)). For *Dominance*, agreement between the two studies was also very high: persevering face (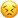), nauseated face (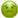), flushed face (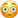), yawning face (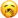) and sleeping face (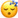) for the most submissive emoji, and collision (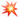), flexed biceps (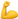), oncoming fist (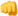) and party popper (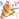) as high in dominance. On this dimension, the largest discrepancy between the two studies was seen for smiling face with sunglasses (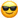). Finally, Figure 2 drew attention to differences in meaning across the 24 emoji, with some being perceived as similar with regard to *Pleasure*, yet different on *Arousal* (e.g., face with steam from nose (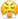) and yawning face (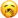)), or how emoji perceived similar with regard to *Dominance* would be associated with the different poles of the *Arousal* dimension (e.g., face screaming in fear (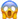) and sleeping face (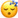)).

**Figure 3:**
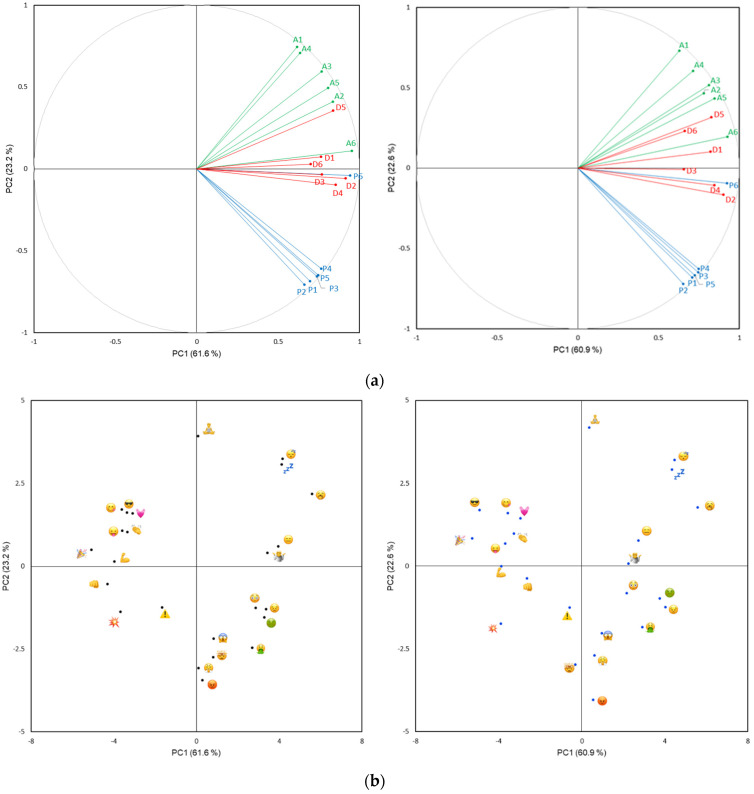
Two-dimensional spaces following Principal Components Analysis for PAD ratings of 24 emoji in Study 1 (NZ, left) and Study 2 (UK, right). (**a**) Variables plot spanned by PC1 and PC2, and (**b**) Observations plot spanned by PC1 and PC2. For variables plot, colors are used to visually highlight the PAD dimensions, with blue for P1 to P6, green for A1 to A6 and red for D1 to D6.

To further support the exploration of emoji meaning according to the PAD model, PCA performed on the 18 PAD variables resulted in three-factor solutions being retained for both studies, accounting for >90% of total variance with ~61%, ~23% and ~8% accounted for by PC1, PC2 and PC3, respectively. Fitting with the univariate results, the spaces spanned by PC1 and PC2 were highly similar and captured variation in each of the *Pleasure*, *Arousal* and *Dominance* dimensions. The continuum from pleasure to displeasure is located on an axis spanning from the 2nd quadrant (top left) to the 4th quadrant (bottom right) (Figure 3, top) and separating smiling face emoji (face with tongue (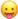), face savouring food (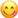) and smiling face with sunglasses (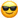)) from those perceived negatively, for example, face vomiting (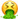), pouting face (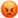) and face with steam from nose (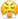) (Figure 3, bottom). The *Arousal* dimension was located approximately perpendicular to the *Pleasure* dimension and spanned from the 3rd quadrant (lower right) to the 1st quadrant (upper left) separating emoji perceived as high in arousal (e.g., oncoming fist (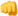), flexed biceps (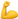) and collision (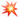)) from those perceived as low in arousal (e.g., yawning face (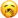), zzz (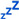) and sleeping face (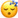)). The *Dominance* dimension ran along PC1 (Figure 3, top) separating high dominance emoji such as oncoming fist (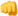) and flexed biceps (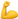) from low dominance (i.e., submissive) emoji-like flushed face (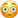) and persevering face (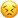) (Figure 3, bottom). Part 7 of Supplementary Materials contains the spaces for variables and observations spanned by PC2 and PC3, where the separation of emoji along the continuum from arousal to nonarousal was clearly seen. The RV coefficients calculated on three dimensions exceeded 0.95 [32] and confirmed the high degree of similarity in Study 1 and Study 2 results: emoji (0.956) and PAD variables (0.974).

### 3.2. Emoji Appropriateness and Use

The results linked to Objective 2 are shown in Table 2. The data for frequency of emoji use (Study 1 only) identified a grouping of five emoji that were most frequently used (face with tongue (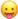), face savouring food (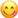), party popper (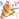), beating heart (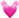) and smiling face with sunglasses (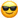)), and significantly more than a group of eight emoji (persevering face (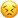), exploding head (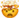), warning (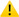), collision (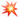), flexed biceps (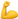), yawning face (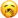) and person in lotus position (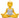)). The average frequency of use for emoji in the former group was between the scale anchors ‘sometimes (not all the time, but neither rarely, 35–65%)’ and ‘infrequently (generally not, but on occasion, 10–35%)’ while the least used emoji, on average, were more likely to be used ‘never or very infrequently (<10%)’. Correlation analysis on the values in Table 2 showed that the emoji-specific use frequencies were statistically associated with perceived appropriateness for use in situations relating to F&Bs or eating and drinking, and as one increased so did the other (r = 0.50 and 0.56, respectively; *p* < 0.015).

Regarding perceived suitability of use of the 24 emoji for F&Bs or situations relating to eating and drinking, the two studies were quite similar (r = 0.82, *p* < 0.001) and for past emoji use in food-related situations the two sets of responses were also positively correlated (r = 0.73, *p* < 0.001). Face savouring food (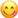), clapping hands (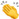) and party popper (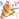) were in the top-5 for both studies, as was a strong negative expression linked to rejection (Study 1: face vomiting (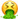); Study 2: nauseated face (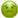)). The same two emoji were regarded as least appropriate in both studies: zzz (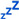) and oncoming fist (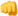).

There appeared to be a systematic difference between the two studies where frequency of selection of emoji as appropriate was higher among participants in Study 1 than in Study 2. Based on 50% citation frequency as the boundary value for “appropriateness”, the emoji collision (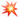), flexed biceps (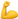) and person in lotus position (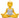) would be regarded as appropriate in Study 1 but not Study 2 (100%, 82% and 82% vs. 42%, 39% and 25%). Among the 24 emoji included in the research, only four did not exceed the 50% criterion in Study 1 (exploding head (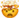), oncoming fist (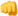), yawning face (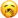) and zzz (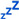)); while only seven emoji exceeded the 50% criterion in Study 2 (face savoring food (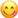), nauseated face (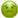), clapping hands (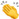), party popper (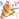), beating heart (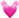), face with tongue (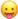) and smiling face with sunglasses (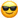)). This systematic difference between the two studies also extended to the responses about the previous use of the focal emoji for F&Bs or situations relating to eating and drinking. In Study 2, stated use linked to F&Bs or eating and drinking situations was above 33% for two emoji only face savouring food (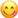) and nauseated face (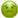). In Study 1, 10 of the 24 emoji exceeded this criterion.

In both studies, participants’ general attitudes to emoji were positive (Table 3), with two exceptions: “I consider myself emoji literate” and “I like to use many different emoji, of all kinds.” For these statements, the average values were closer to the scale anchor ‘neither agree nor disagree’ and the standard deviations were larger. The latter pointed to heterogeneity, which histograms of response distributions confirmed. In fact, an exploratory hierarchical cluster analysis (Euclidean distance, Ward’s method) based on these attitudinal statements established two consumer segments (Part 8 of Supplementary Material) where participants in one of the segments agreed much more strongly that emoji were best suited to private communications, and they described themselves as less emoji savvy and less likely to use many different emoji. The two clusters did, however, not differ with regard to the agreement on the statements that emoji were fun to use and a means to express moods/emotions and oneself more generally.

## 4. Discussion

### 4.1. Emoji Meanings

A lack of knowledge about their meaning makes in-depth interpretation of results from food-related consumer research that involves emoji more challenging. Against this background, the present research established emoji meanings for 24 emoji on the *Pleasure, Arousal* and *Dominance* (PAD) dimensions, and characterisation of individual emoji against the 18 pairs of semantic differentials and three dimensions was the primary contribution of the research (Figure 2 and Figure 3; Parts 4 to 7 of Supplementary Materials). The finding that differences in the meaning of the 24 emoji were greatest on the *Pleasure* dimension followed by the *Arousal* dimension (Figure 2, Parts 5 and 6 of Supplementary Material) fit with past empirical research [23,33], and the focus in emotion research on these two dimensions as the key determinants of human affect [17]. It provided information to aid researchers in interpreting consumers’ emoji use, be it in questionnaires that capture associations to F&Bs or on social media where emoji use is prevalent both in relation to situations involving eating and drinking and more generally [6,28,34].

In relation to the concern expressed by Jaeger, Jin et al. [35] that the emoji typically used in food-related consumer research may be less able to span the two-dimensional valence × arousal space compared to emotion words, the findings from the present research show that it is possible to identify emoji that span this space (e.g., face vomiting (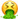), face savouring food (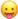), sleeping face (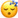) and collision (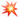)). Moreover, the positions of emoji in the space spanned by the first two components following PCA of the 19 PAD variables (Figure 3) were in line with prior research suggesting that emoji can span the valence × arousal configuration [9]. Their ability to do so is directly linked to their selection, and in this regard, emoji do not differ from emotion words. For example, the EsSense Profile™ [36] is dominated by words with positive valence and limited variation in arousal and as a result it poorly spans the full valence × arousal space [37].

The larger span in average scores on the *Pleasure* and *Arousal* dimensions than on the *Dominance* dimension meant that the 24 emoji differed least on the dominance to submissive continuum. While possibly an artefact of emoji selection, this result seemed to align well with the *Dominance* being given less consideration in emotion research [17] and imply that *Dominance* supplements emoji meanings rather than being a critical aspect hereof. The average scores on this dimension fitted expectations with oncoming fist (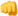) and flexed biceps (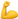) as representatives of high dominance, and flushed face (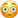) and person shrugging (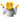) as representatives of submission.

As recommended by Jaeger, Vidal and Ares [4], the research included several emoji that until now have not been used in the context of F&B research, and many of these were non-facial (e.g., 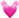, 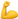, 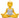). Early research with emoji tended to focus on facial emoji because facial expressions are critical to understanding essential human emotions in face-to-face communication [38]. However, the finding that certain non-facial emoji contributed nuance in the expression of pleasure/positive valence (Figure 3, Part 5 of Supplementary Material) was interesting and suggested that they may contribute to a more complete representation of the core dimensions of human affect [22]. Prior research has found out that certain non-facial emoji are frequently used by consumers to describe their emotions in a food consumption context and have a high discriminative ability [10,39]. We recommend that non-facial be considered for inclusion in research, especially where it concerns F&B products or situations that vary in degree of positive valence. The results from the general emoji attitudes (Table 3) support this in the sense that there did not appear to be a strong preference for facial emoji.

Use of the PAD scale to establish emoji meanings was successful and there was good agreement between the average PAD scores of this study and the scores for valence and arousal obtained by Jaeger, Roigard et al. [22] using Self-Assessment Manikins (SAM: Bradley and Lang [21]). Since each PAD dimension is an average of six semantic differential, this measurement approach requires more effort than the SAMs, but it may be worthwhile considering that the manikins are not intuitive to everybody [40]. The use of the three different PAD dimensions and 7-point scale has the advantage that it offers a more nuanced perspective compared to traditional sentiment analysis which generally only provides a three-way classification of positive, neutral and negative sentiments [41]. A limitation of PAD and SAM approaches, however, is that they do not provide detailed semantic meanings. For example, persevering face (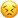) and nauseated face (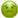), had about the same average scores on the PAD dimensions (Part 6 of Supplementary Material), with a high degree of displeasure, medium arousal, and high dominance. Yet, they mean different things, and this was not adequately captured. Therefore, emoji meanings on PAD dimensions should not be regarded as stand-alone as they are not comprehensive. However, as shown by Jaeger, Roigard et al. [22] combining SAM and textual descriptions to explore the meanings of facial emoji is useful, and neither is complete without the other.

Cross-cultural comparison was not a primary research aim, but the similarity in results obtained from adult participants in NZ and UK was noteworthy nonetheless and contributed new empirical evidence to underpin the viewpoint that the interpretation of emoji is very similar across different cultures [4]. The results also aligned with another recent study where similar emoji meanings were found among adult consumers from the USA and China [23] and further supported the suitability of emoji for cross-cultural research.

### 4.2. Emoji Appropriateness for Use in Research Linked to F&Bs and Eating/Drinking

In addition to establishing emoji meanings on the PAD dimensions, the present research considered the appropriateness of the 24 emoji for use with F&Bs and for situations involving eating and drinking (Obj. 2). It appeared that the results from the two studies differed considerably since a cut-off value of 50% citation frequency for appropriateness meant that 20 out of 24 emoji were considered as appropriate in Study 1 (NZ) while only 7 out of 24 emoji were regarded as appropriate in an F&B context by the participants in Study 2 (UK). While this at a first glance suggested that cultural differences could largely influence perceived emoji appropriateness, a more likely explanation for the differences in citation frequency for appropriateness of use was that data collection in NZ took place in a CLT setting while data collection in the UK was via an online survey. It could be that the participants at a CLT are more motivated or involved (e.g., they already make an effort to come to the CLT) while participants at an online survey might be more driven by incentives. This suggestion takes into consideration the strong positive correlation between the NZ and UK data for appropriateness (r = 0.82) and the fact that question format has a big impact on citation frequency. In a methodological study that compared five question formats for emoji research [9], Ares and Jaeger showed that forced yes/no questions resulted in a higher citation for individual emoji compared to CATA questions but not greater sample discrimination. Assuming this difference has to do with greater effort and attention required for yes/no questions, it could explain why responses from the NZ participants in a CLT setting was characterised by higher citation frequencies but a similar relative ordering of the most and least suitable emoji. Therefore, it may not be appropriate to apply a fixed cut-off value as the main criterion for whether an emoji is regarded as suitable or not. Moreover, the value used here 50% seems conservative considering the recent work by Sick et al. [8] who examined the appropriateness of 92 facial emoji using different eating contexts (e.g., breakfast, snack, birthday) and deemed that emoji which were selected by at least 20% of the participants in at least one eating context qualified as appropriate.

What this further suggests is that decisions about emoji selection should be study specific. This would contribute flexibility to address situations and topics that consumers may not have readily considered when asked to rate appropriateness. It is possible to imagine product-focused research into beverages that help people get a good night’s sleep where 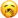 and 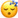 would be appropriate, development of spicy-hot cooking sauces where 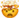 would be appropriate or that 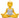 could have relevance in describing tranquil situations such as sitting in the back garden with a glass of wine enjoying the sunset. Other examples include the consumption of insects or gene-edited foods, where certain emoji become relevant even though they would not be regarded as appropriate in the context of typical eating situations.

Participants’ general attitudes to emoji were positive, but there was evidence of segmentation that could have influenced the perceived appropriateness of emoji use in the F&B context. It seemed that this segmentation was not country specific but differentiated between participants who found emoji less suitable for professional use and relied on a smaller set of emoji in computer-mediated communications. Thus, despite the widespread adoption and popularity of emoji [42,43], some people are more positive and frequent users. A study by Jaeger, Xia et al. [15] found that frequency of emoji use did not influence research participants ability to use emoji questionnaires to characterise F&B stimuli and it could be that despite differences in emoji attitude and personal use, research participants are similarly able to use emoji for expressing their emotional and conceptual associations to F&Bs and situations involving eating and drinking.

### 4.3. Limitations and Suggestions for Future Research

Limitations and suggestions for future research pertain to both objectives of the present research. The NZ study was carried out as a CLT while the data was collected online in the UK study due to budget restrictions. As such, data of less participants were collected during the NZ study so one needs to consider the NZ results as more preliminary data. Further, given the different testing’s conditions, one need to be careful about a direct comparison of the NZ and UK results. With regard to emoji meaning (Obj. 1), the PAD model [25] can be seen as an alternative to SAM [21] for gaining insights about emoji meanings, although both should be combined with semantic information (e.g., through open questions as done in Jaeger, Roigard et al. [22]) to obtain a full understanding of the emoji meanings. We expect that a global study combining such information sources across many emoji would enhance their uptake in F&B research, especially if also linked to the appropriateness of emoji use in the F&B context. With regard to the latter, an interesting extension would be to examine the similarity between the perceived appropriateness indicated by consumers and their actual use of emoji in real life, hereby linking this work to past research characterising emoji use in the F&B context, including tweets [28] and restaurant reviews [44]. More generally, future research should examine the interplay between the meanings of emoji and their appropriateness in the F&B context. This is necessary considering that context is crucial for the meanings attached to emoji.

The perception that emoji are only suitable for research with younger persons has been suggested as a barrier to uptake of emoji [4]; and while beyond the scope of this study to examine potential age effects regarding emoji meaning and appropriateness, this is a relevant topic for future research. A recent study found that emoji might be differently understood depending on age and gender, showing, for example, that respondents aged 30 years and above tended to interpret emoji more literally while younger users interpreted them in more conventionalized ways [45]. Emoji also tend to be more frequently used by women than men [42], but more in private communication than public communication [46]. This study only included data from people who used emoji at least infrequently so it is relevant that future research includes comparisons between participants who differ in emoji usage (e.g., non-users, vs. regular users vs. superusers).

A better understanding of the meaning and appropriateness of emoji in the F&B context also has relevance and value for product development and marketing. For example, the inclusion of emoji in advertisements leads to higher purchase intentions and positive affect [47], and an analysis of the four largest Spanish beer companies revealed how emoji were used as a differentiating element for brand positioning [48]. Against this backdrop, it would be interesting to examine if emoji can be used in the same way as emotional/conceptual profiling to strengthen the product experience when the emotional message by the product is consonant with the brand [49]. In line with this, further research could examine the sensory drivers of product emotions which has been established using emotion words [50] but not emoji.

## 5. Conclusions

Emoji meanings and appropriateness for use in F&B research were investigated in the present research. A total of 24 emoji were considered, which included several non-facial emoji that had not been previously investigated. The research also extended past studies by including consumers from NZ and UK and hereby extending the geographical reach of empirical research into emoji meanings. The PAD model *Pleasure, Arousal, Dominance* was used to establish emoji meanings and this dimensional approach was successful in characterizing and differentiating between the included emoji. The biggest differences in meaning were for *Pleasure*, followed by *Arousal*, and based on the included emoji it was possible to identify a subset that spanned the valence × arousal space well. *Dominance* was, on average, the least important of the PAD dimensions for characterizing emoji meaning. The results from NZ and UK were remarkably similar with regard to emoji meanings, in support of past claims that emoji have high suitability for cross-cultural research. Regarding the appropriateness of emoji for use in F&B research, similarity in NZ and UK results remained quite high. Values for perceived appropriateness were established, but we leave it to individual researchers to decide which threshold they use as the cut-off regarding whether a certain emoji is suitable for a particular research activity.

## Figures and Tables

**Table 1:** The 18 semantic differentials in the pleasure-arousal-dominance (PAD) scale used in Study 1 and Study 2, with end-point anchors shown.

Dimension	PAD Code	Left Anchor (1)	Right Anchor (9)
**Pleasure** (Pleasure- Displeasure)	P1	Happy	Unhappy
	P2	Pleased	Annoyed
	P3	Satisfied	Unsatisfied
	P4	Contented	Melancholic
	P5	Hopeful	Despairing
	P6	Amused *	Bored
**Arousal** (Arousal- Nonarousal)	A1	Stimulated	Relaxed
	A2	Excited	Calm
	A3	Frenzied	Sluggish
	A4	Jittery	Dull
	A5	Wide-awake	Sleepy
	A6	Aroused	Unaroused
**Dominance** (Dominance- Submissiveness)	D1	Controlling	Controlled
	D2	Influential	Influenced
	D3	In control	Cared for
	D4	Important	Awed
	D5	Dominant	Submissive
	D6	Autonomous	Guided

Note. * Changed scale anchor relative to Mehrabian and Russell (1974), in accordance with Detandt, Leys [30].

**Table 2:** Emoji use characteristics among participants in the two studies. In Study 1 (NZ) each emoji was evaluated by 40 to 43 participants and in Study 2 (UK) each emoji was evaluated by 99 to 116 participants.

		Study 1 (NZ)			Study 2 (UK)	
Emoji Name and Image		Avg. Frequency of Using This Emoji *	Emoji Use in F&B or Eating Situation Is Appropriate (%)	Have Used Emoji in F&B or Eating Situation (%)	Emoji Use in F&B or Eating Situation Is Appropriate (%)	Have Used Emoji in F&B or Eating Situation (%)
Beating heart	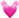	2.7 ^ef^	69	38	56	18
Clapping hands	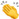	2.3 ^bcdef^	88	33	56	22
Collision	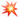	1.6 ^abc^	77	19	42	16
Exploding head	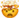	1.7 ^abc^	43	7	43	18
Expressionless face	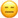	2.1 ^abcdef^	60	20	33	13
Face savouring food	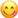	2.8 ^ef^	86	49	75	39
Face screaming in fear	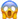	2.3 ^bcdef^	63	35	44	20
Face vomiting	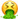	1.8 ^abcd^	72	49	40	22
Face with steam from nose	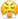	1.7 ^abc^	43	10	32	11
Face with tongue	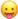	2.9 ^f^	71	29	55	25
Flexed biceps	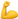	1.8 ^abc^	65	15	39	12
Flushed face	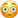	2.4 ^cdef^	63	35	34	18
Nauseated face	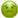	2.1 ^abcdef^	69	38	60	34
Oncoming fist	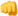	2.2 ^abcdef^	38	20	25	14
Party popper	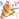	2.7 ^ef^	78	43	56	26
Persevering face	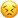	1.8 ^abc^	50	8	31	15
Person in lotus position	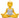	1.4 ^a^	49	14	25	7
Person shrugging	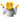	2.2 ^bcdef^	52	21	33	15
Pouting face	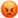	2.0 ^abcde^	48	30	39	12
Sleeping face	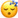	2.4 ^cdef^	50	15	27	9
Smiling face with sunglasses	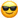	2.7 ^def^	73	18	50	25
Warning	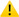	1.6 ^ab^	50	10	25	11
Yawning face	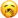	1.8 ^abc^	48	13	28	12
Zzz	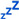	2.1 ^abcdef^	36	7	25	10

Notes. F&B = food and beverage. * Stated emoji use frequency measured on a 5-pt scale with the following anchors: 5 = ‘Always or almost every time (>90%)’, 4 = ‘Frequently (more often than not, 65–90%)’, 3 = ‘Sometimes (not all the time, but neither rarely, 35–65%)’, 2 = ‘Infrequently (generally not, but on occasion, 10–35%)’ and 1 = ‘Never or very infrequently (<10%)’.*) Post hoc test performed using Tukey’s HSD and emoji that share a letter are not significantly different at the 5% level. This set of data was only collected in Study 1.

**Table 3:** Average score * (M) and standard deviation (SD) for general emoji attitudes among participants. Shown for Study 1 (NZ: *n* = 165 and Study 2 (UK: *n* = 861).

Statement	Study 1 (NZ)		Study 2 (UK)	
	M	SD	M	SD
Emoji are fun to use and receive	5.7	1.2	5.3	1.3
Emoji are more appropriate in private than professional communications	6.0	1.3	5.8	1.3
I consider myself to be emoji savvy and literate	4.5	1.7	4.3	1.7
I generally use a small set of emoji over and over again	6.0	1.0	5.4	1.3
I like to use many different emoji, of all kinds	4.3	1.8	4.4	1.7
In computer-mediated communications, emoji help me to better express myself	5.3	1.4	4.8	1.5
My favourite emoji are “face emoji” (e.g., 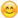 , 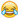 , 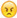 )	5.8	1.3	5.3	1.3
Using emoji helps me to express my moods/emotions	5.9	1.2	5.1	1.4

Notes. * 1 = Disagree Strongly; 2 = Disagree Moderately; 3 = Disagree Slightly; 4 = Neither agree nor disagree; 5 = Agree Slightly; 6 = Agree Moderately; 7 = Agree Strongly.

## Data Availability

The data presented in this study are available on request from the corresponding author.

## Supplementary Materials

The following are available online at https://www.mdpi.com/article/10.3390/foods10112880/s1, (1) Summary of participant characteristics; (2) Exemplar ballots for PAD scales; (3) Emoji allocation to participants; (4) Means for 18 PAD variables on 24 emoji with Tukey HSD results; (5) Line plots of means for 18 PAD variables on 24 emoji; (6) Means for 3 PAD dimensions on 24 emoji; (7) Spaces for PAD variables spanned by PC2 and PC3 following PCA; (8) Cluster analysis on general emoji attitude data.
