# Supplementary material for: Emoji for Food and Beverage Research: Pleasure, Arousal and Dominance Meanings and Appropriateness for Use
Source: Foods. 2021 Nov 22;10(11):2880. doi: 10.3390/foods10112880 (PMC8619849; doi:10.3390/foods10112880)

SUPPLEMENTARY MATERIALS

Part 1a of Supplementary Material

Participant characteristics for Study 1 (NZ) shown as percentage (%) unless otherwise indicated.

	All participants (n=180)	Emoji users* (n=165)
Gender		
Female	50	47
Male	50	53
Age		
18-30 y.o.	16	18
31-45 y.o.	34	38
46-65 y.o.	49	45
*Ethnicity***		
NZ European	62	61
Maori	6	7
Pacific Island	6	6
European	8	8
Chinese	10	11
Japanese	1	1
Indian	13	13
Other[1]	9	9
*Household size***		
Alone	13	12
Spouse	60	59
Child under 18	40	41
Child over 18	13	12
Parents	12	13
Flatmate	14	15
Other[2]	5	5
Household size		
1 – 2	35	35
3 – 4	47	47
5+	18	18
Household income (NZD)		
Less than $30,000	4	4
$30,000 - $49,999	7	7
$50,000 - $79,999	20	21
$80,000 - $99,999	12	11

$100,000 - $119,999	16	16
$120,000 or more	34	34
Prefer not to answer	7	6

Notes: *) Participants who use emoji at least infrequently (>10%) in their messages and social media conversations. **) Total responses are >100% as consumers may select multiple options. [1]) Other ethnicities included African, Filipino, Indonesian, Korean, Latin American, Malaysian, Maltese, and South African. [2]) Other household members included cousin, grandparents, in-laws, nephew, and siblings.

Part 1b of Supplementary Material

Participant characteristics for Study 2 (UK) shown as percentage (%) unless otherwise indicated.

	All participants (n=1047)	Emoji users* (n=861)
Gender		
Female	50	46
Male	50	54
Age		
18-30 y.o.	14	17
31-45 y.o.	35	39
46-65 y.o.	51	44
*Ethnicity***		
British	85	83
Chinese	1	2
Indian	2	2
Pakistani	2	2
Black African	2	2
Black Caribbean	1	2
Other[1]	9	10
*Household member***		
Alone	22	20
Spouse	57	57
Child under 18	29	31
Child over 18	11	11
Parents	13	13
Flatmate	3	3
Other[2]	3	4
Household size		
1 – 2	55	52
3 – 4	39	41
5+	6	7
Household income (GBP)		
Less than £10,000	7	6
£10,000-£19,999	16	15
£20,000-£29,999	18	18
£30,000-£39,999	17	18
£40,000-£49,999	13	13
£50,000 or more	24	25
Prefer not to answer	5	5

Notes: *) Participants who use emoji at least infrequently (>10%) in their messages and social media conversations. **) Total responses are >100% as consumers may select multiple options. [1]) Other

ethnicities included American, Australian, Canadian, European, Filipino, Japanese, Latin American, Middle Easterner, mixed background, Nepalese, Russian, South African, and prefer not to answer. [2]) Other household members included cousin, grandparents, in-laws, siblings, and prefer not to answer

Part 2 of Supplementary Material

2a. Exemplar ballot for PAD scales used in Study 1 (NZ)

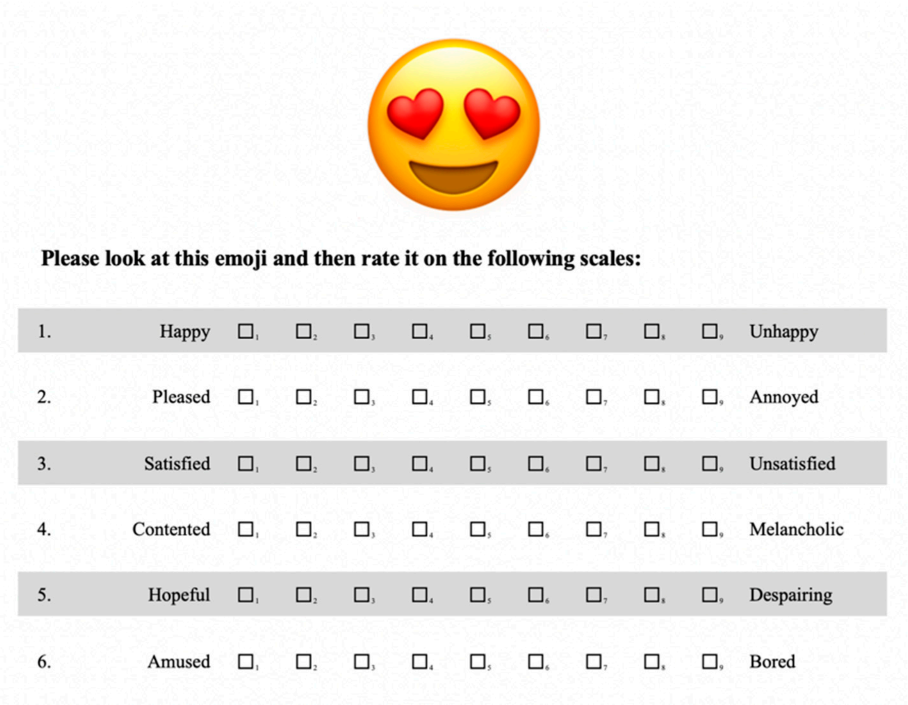

Please look at this emoji and then rate it on the following scales:

1.	Happy	☐1	☐2	☐3	☐4	☐5	☐6	☐7	☐8	☐9	Unhappy
2.	Pleased	☐1	☐2	☐3	☐4	☐5	☐6	☐7	☐8	☐9	Annoyed
3.	Satisfied	☐1	☐2	☐3	☐4	☐5	☐6	☐7	☐8	☐9	Unsatisfied
4.	Contented	☐1	☐2	☐3	☐4	☐5	☐6	☐7	☐8	☐9	Melancholic
5.	Hopeful	☐1	☐2	☐3	☐4	☐5	☐6	☐7	☐8	☐9	Despairing
6.	Amused	☐1	☐2	☐3	☐4	☐5	☐6	☐7	☐8	☐9	Bored

2b. PAD scale screenshot from Study 2 (UK)

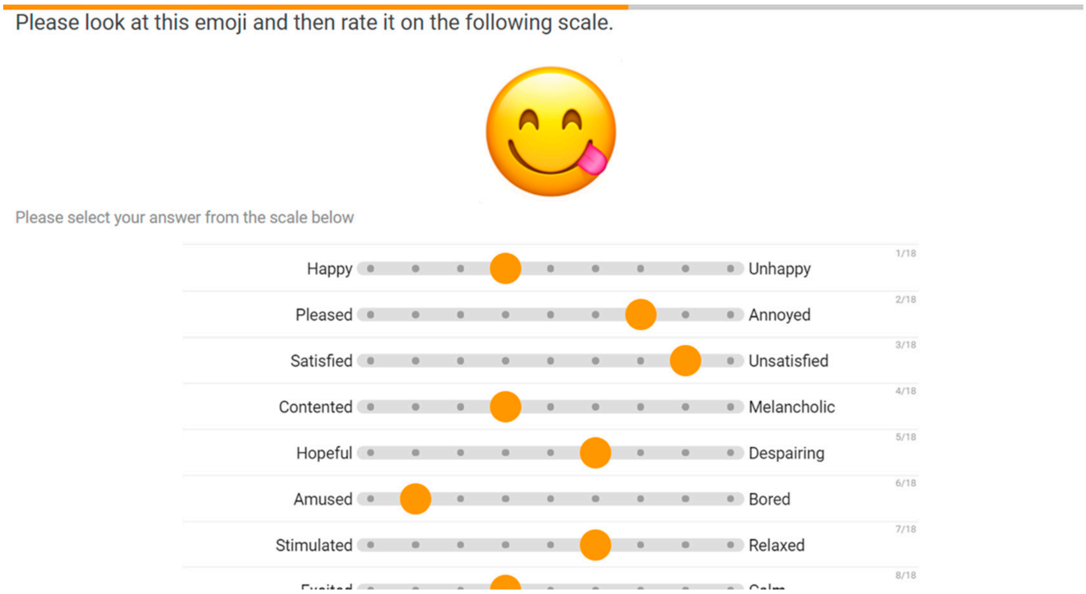

Part 3 of Supplementary Material

3a. Emoji allocation in Study 1 (NZ)
Each participant in Study 1 evaluated six emoji and participants were assigned to one of the 4 groups shown below, which collectively covered the 24 emoji included in the study.

Group 1		Group 2		Group 3		Group 4	
Collision	💥	Beating heart	💓	Clapping hands	👏	Expressionless face	😑
Face savoring food	😋	Exploding head	🤯	Flexed biceps	💪	Face with steam from nose	😤
Face screaming in fear	😱	Nauseated face	🤢	Pouting face	😡	Oncoming fist	👊
Face vomiting	🤮	Person shrugging	🤷	Warning	⚠️	Party popper	🎉
Person in lotus position	🧘	Zzz	💤	Yawning face	🥱	Sleeping face	😴
Flushed face	😳	Face with tongue	😛	Smiling face with sunglasses	😎	Persevering face	😣

3b. Emoji allocation in Study 2 (UK)
Each person completed the task for 3 of the 24 emoji (full listing in Table 1 in Main paper), and the allocation of emoji to participants were such that each participant evaluated 3 different emoji, and each emoji were evaluated by the same proportion of participants in four groups defined as younger men, older men, younger women and older women (where the younger age group was 18 to 45 years old and the older age group was 46 to 65 years old).

Part 4a of Supplementary Material

Averages* for the 18 PAD scales in Study 1 (NZ) (P1 to P6 for *Pleasure* (P), A1 to A6 for *Arousa*l (A) and D1 to D6 for *Dominance*). Generally: for P dimension, the low anchor (1) is associated with pleasure and the high anchor (9) is associated with displeasure; for the A dimension, the low anchor (1) is associated with arousal and the high anchor (9) is associated with non-arousal; for the D dimension, the low anchor (1) is associated with dominance and the high anchor (9) is associated with submissiveness. Table 1 in the main text has the full set of scale anchors.

Emoji name	P1	P2	P3	P4	P5	P6	A1	A2	A3	A4	A5	A6	D1	D2	D3	D4	D5	D6
Beating heart	1.7<br>l	2.0<br>jk	2.1<br>hij	2.1<br>j	2.0<br>l	2.9<br>jk	4.0<br>cde	2.5<br>fgh	3.7<br>ghi	4.2<br>ef	3.1<br>efgh	2.8<br>jkl	4.5<br>bcdefg	4.0<br>defg	5.6<br>ab	3.3<br>gh	4.4<br>cdef	4.9<br>abc
Clapping hands	2.0<br>i	1.6<br>k	2.3<br>hij	2.6<br>hij	2.7<br>kl	2.7<br>jk	2.9<br>def	2.2<br>gh	3.5<br>ghi	4.2<br>ef	2.4<br>fgh	3.2<br>jkl	4.8<br>abcdef	4.2<br>cdefg	4.0<br>cdefghi	4.2<br>cdefg	4.5<br>bcdef	4.5<br>abc
Collision	4.1<br>g	4.9<br>fgh	4.0<br>fg	4.2<br>efg	4.2<br>hij	3.3<br>ijk	2.0<br>f	2.2<br>gh	2.9<br>hi	3.1<br>f	2.1<br>gh	3.2<br>ijkl	4.0<br>defg	3.7<br>efg	4.0<br>defghi	3.6<br>fgh	2.5<br>ghi	4.0<br>bc
Exploding head	7.5<br>abcd	7.3<br>bcd	7.2<br>abc	5.7<br>abcd	7.1<br>abcd	4.8<br>fgh	2.9<br>def	3.4<br>defg	2.8<br>hi	3.3<br>f	2.9<br>efgh	5.4<br>defg	5.1<br>abcde	5.4<br>abcd	5.3<br>abcde	5.0<br>abcdef	4.0<br>defg	5.1<br>abc
Expressionless f.	6.8<br>bcde	6.9<br>cde	7.2<br>abc	5.7<br>abcd	6.7<br>abcde	7.1<br>abc	5.6<br>b	5.6<br>b	6.7<br>ab	6.8<br>ab	5.9<br>b	7.0<br>abcd	5.7<br>abc	5.8<br>ab	5.1<br>abcdef	5.2<br>abcde	5.9<br>abc	4.9<br>abc
F. savoring food	1.4<br>i	1.7<br>k	1.7<br>j	1.9<br>j	2.4<br>l	2.5<br>jk	4.0<br>cde	2.4<br>fgh	3.9<br>fghi	4.5<br>cdef	2.6<br>efgh	2.6<br>kl	4.8<br>abcdef	3.9<br>defg	4.3<br>abcdefgh	3.8<br>efgh	3.9<br>defg	4.5<br>abc
F. screaming in fear	6.6<br>cde	6.6<br>cde	6.7<br>bcd	6.0<br>abc	7.0<br>abcd	4.6<br>ghi	3.0<br>def	3.3<br>defg	3.4<br>ghi	3.5<br>ef	3.2<br>efgh	4.3<br>ghij	6.1<br>a	5.5<br>abcd	4.6<br>abcdefg	5.4<br>abc	4.8<br>abcde	4.8<br>abc
F. vomiting	8.3<br>a	7.9<br>abc	8.3<br>a	6.9<br>a	7.8<br>ab	6.1<br>bcdef	3.2<br>def	4.7<br>bcd	4.0<br>efgh	4.1<br>ef	4.0<br>cde	6.0<br>bcdef	6.0<br>ab	5.0<br>bcdef	5.5<br>abcd	5.6<br>abc	5.0<br>abcd	5.0<br>abc
F. with steam from nose	8.5<br>a	8.5<br>ab	8.0<br>ab	6.0<br>abc	7.2<br>abcd	5.6<br>defg	3.0<br>def	3.7<br>cdef	3.5<br>ghi	3.4<br>f	3.3<br>defgh	4.1<br>ghijk	4.0<br>defg	5.1<br>abcde	4.2<br>bcdefghi	4.5<br>bcdefg	3.6<br>defgh	4.4<br>abc
F. with tongue	1.7<br>i	2.0<br>jk	2.7<br>ghij	2.5<br>hij	2.7<br>kl	2.0<br>k	3.1<br>def	2.5<br>fgh	3.8<br>fghi	4.2<br>ef	2.1<br>gh	3.4<br>hijkl	4.4<br>bcdefg	4.2<br>cdefg	3.6<br>fghi	3.9<br>efgh	4.1<br>def	4.6<br>abc
Flexed biceps	3.5<br>gh	3.3<br>ij	3.2<br>ghi	3.5<br>ghi	3.4<br>jkl	3.6<br>hij	2.8<br>def	3.2<br>efg	3.6<br>ghi	4.5<br>cdef	2.8<br>efgh	3.5<br>hijkl	3.2<br>fg	3.4<br>fg	2.7<br>i	3.7<br>efgh	2.3<br>hi	4.0<br>bc
Flushed f.	6.6<br>cde	6.2<br>def	6.8<br>bcd	6.1<br>abc	6.5<br>bcdef	5.4<br>efg	3.5<br>def	4.6<br>bcd	4.4<br>defg	4.4<br>cdef	3.8<br>cdef	5.0<br>efgh	6.3<br>a	6.6<br>a	5.6<br>ab	6.2<br>a	5.9<br>abc	5.3<br>abc
Nauseated f.	8.1<br>ab	7.2<br>bcd	8.0<br>ab	6.8<br>a	7.3<br>abc	5.8<br>bcdefg	3.8<br>cde	5.0<br>bc	5.4<br>bcde	4.5<br>cdef	4.0<br>cde	6.6<br>abcde	5.9<br>abc	5.5<br>abcd	5.8<br>a	5.4<br>abcd	6.0<br>ab	5.1<br>abc
Oncoming fist	4.1<br>g	4.0<br>hi	3.6<br>fgh	3.7<br>fgh	4.0<br>ijk	3.7<br>hij	2.8<br>def	2.4<br>fgh	3.4<br>ghi	4.1<br>ef	2.5<br>fgh	3.3<br>ijkl	3.0<br>g	3.3<br>g	2.7<br>i	3.5<br>gh	2.0<br>i	3.8<br>c
Party popper	1.3<br>i	1.8<br>k	1.9<br>ij	2.7<br>hij	2.2<br>l	2.1<br>k	2.2<br>f	1.6<br>h	2.5<br>i	3.7<br>ef	1.9<br>h	2.1<br>l	4.3<br>cdefg	3.6<br>efg	3.9<br>efghi	3.5<br>gh	3.3<br>efghi	4.2<br>abc
Persevering f.	7.7<br>abc	7.3<br>bcd	8.0<br>ab	6.4<br>abc	8.0<br>a	6.2<br>bcde	4.2<br>bcd	4.7<br>bcd	5.2<br>cdef	5.0<br>cde	5.2<br>bc	5.7<br>cdefg	6.0<br>ab	5.9<br>ab	5.3<br>abcde	5.4<br>abcd	6.0<br>a	4.9<br>abc
Person in lotus position	2.5<br>hi	2.5<br>jk	2.2<br>hij	2.5<br>hij	2.5<br>l	4.8<br>fgh	7.8<br>a	7.4<br>a	5.9<br>bc	5.8<br>bc	5.9<br>b	4.8<br>fghi	4.7<br>abcdef	4.6<br>bcdefg	3.0<br>hi	4.4<br>bcdefg	5.7<br>abc	5.2<br>abc
Person shrugging	5.9<br>ef	5.8<br>efg	6.1<br>cde	6.0<br>abc	5.9<br>defg	6.0<br>bcdef	5.1<br>bc	5.5<br>b	5.8<br>bcd	5.8<br>bcd	4.7<br>bcd	6.4<br>abcdef	6.0<br>ab	5.7<br>abc	5.5<br>abc	5.7<br>ab	6.1<br>a	5.6<br>a
Pouting f	8.7<br>a	8.8<br>a	8.4<br>a	6.5<br>ab	7.5<br>abc	5.7<br>cdefg	2.5<br>ef	4.3<br>bcde	3.4<br>ghi	3.3<br>f	3.4<br>defg	4.8<br>fghi	3.7<br>efg	4.8<br>bcdefg	4.2<br>abcdefghi	4.3<br>bcdefg	3.2<br>fghi	4.7<br>abc
Sleeping f.	4.7<br>fg	4.6<br>ghi	4.9<br>ef	4.4<br>defg	5.1<br>ghi	7.2<br>ab	8.1<br>a	7.9<br>a	7.5<br>a	6.9<br>ab	8.5<br>a	7.5<br>ab	5.5<br>abcd	6.0<br>ab	5.7<br>ab	5.2<br>abcde	6.1<br>a	4.8<br>abc
Smiling f. with sunglasses	1.9<br>i	1.9<br>jk	2.2<br>hij	2.2<br>ij	2.5<br>l	2.6<br>jk	4.3<br>bcd	3.4<br>defg	4.0<br>efgh	4.3<br>def	2.7<br>efgh	3.4<br>hijkl	4.8<br>abcdef	4.2<br>cdefg	3.7<br>fghi	4.0<br>defgh	4.0<br>defg	4.1<br>abc

Warning	5.7 ef	6.0 def	5.7 de	5.3 bcde	5.4 efgh	4.8 efgh	3.2 def	3.3 defg	3.6 ghi	4.2 ef	3.1 efgh	4.1 ghijk	3.8 efg	3.6 efg	3.4 ghi	2.5 h	3.3 efghi	5.5 ab
Yawning f.	6.3 de	5.8 efg	6.8 bcd	6.2 abc	6.3 cdefg	8.1 a	7.7 a	7.2 a	7.6 a	7.5 a	8.5 a	8.0 a	5.8 abc	5.8 abc	5.7 ab	5.7 abc	6.2 a	5.3 abc
Zzz	4.8 fg	5.0 fgh	5.0 ef	5.0 cdef	5.2 fghi	6.9 abcd	7.8 a	7.8 a	7.6 a	7.2 ab	8.4 a	7.1 abc	5.5 abcd	5.5 abcd	5.5 abc	5.2 abcde	6.1 a	4.9 abc

Notes. *) Tukey's HSD used for post-hoc tests. Within columns, emoji that share a letter are not significantly different at the 5% level.

Part 4b of Supplementary Material

Averages* for the 18 PAD scales in Study 2 (UK) (P1 to P6 for *Pleasure* (P), A1 to A6 for *Arousa*l (A) and D1 to D6 for *Dominance*). Generally: for P dimension, the low anchor (1) is associated with pleasure and the high anchor (9) is associated with displeasure; for the A dimension, the low anchor (1) is associated with arousal and the high anchor (9) is associated with non-arousal; for the D dimension, the low anchor (1) is associated with dominance and the high anchor (9) is associated with submissiveness. Table 1 in the main text has the full set of scale anchors.

Emoji name	P1	P2	P3	P4	P5	P6	A1	A2	A3	A4	A5	A6	D1	D2	D3	D4	D5	D6
Beating heart	2.6 i	2.9 jk	3.0 kl	3.4 hij	3.1 i	3.5 klm	4.5 cd	3.9 hijk	4.4 fg	4.4 ghij	3.5 hijkl	3.8 k	4.9 bcdefg	4.5 efghi	5.5 a	4.7 bcdef	4.6 cdef	4.8 abc
Clapping hands	2.9 i	3.3 ij	3.0 kl	3.8 ghi	3.5 hi	3.8 kl	4.0 defg	3.3 jkl	4.2 g	4.7 efghi	3.4 hijkl	4.6 fghijk	4.9 bcdefg	4.2 ghi	4.4 bcde	4.4 def	4.2 fg	4.9 abc
Collision	4.4 gh	5.1 fg	4.3 ij	4.8 def	4.7 fg	4.0 jk	3.2 g	3.1 kl	3.1 h	3.8 ij	3.0 kl	4.3 ghijk	4.3 fg	4.3 fghi	4.4 cde	4.3 ef	3.3 h	4.4 c
Exploding head	6.5 cd	6.3 bcde	6.2 def	5.6 abcd	6.6 abcd	4.7 hij	3.4 fg	3.9 ijk	3.1 h	3.7 j	3.3 ijkl	4.8 fghij	5.2 abcde	5.3 abcde	4.8 abc	5.1 abcde	4.7 cdef	4.7 abc
Expressionless f.	5.6 def	5.7 defg	5.9 fgh	5.5 abcde	5.7 de	6.2 bcd	5.5 b	6.1 cd	6.1 bc	6.1 bc	5.8 b	6.4 abc	5.5 abcd	5.1 abcdef	4.8 abc	5.1 abcde	5.2 abcde	4.8 abc
F. Savoring food	2.2 i	2.4 k	2.7 l	2.9 ij	3.3 hi	2.9 lm	4.0 defg	3.3 jkl	4.1 g	4.5 fghij	3.2 ijkl	4.0 ijk	4.9 bcdefg	4.7 bcdefgh	4.5 bcd	4.5 cdef	4.5 def	5.1 abc
F. Screaming in fear	6.5 cde	6.0 cdef	6.3 cdef	5.6 abcd	6.7 abc	5.0 fghi	4.0 defg	4.1 hij	4.3 fg	4.2 hij	4.1 efghi	5.1 efgh	5.3 abcde	5.4 abcd	4.9 abc	5.3 abc	5.1 bcde	4.9 abc
F. Vomiting	7.4 ab	7.0 b	7.5 ab	6.2 ab	7.0 a	5.8 cdefg	4.4 cde	5.0 efg	5.5 cd	5.0 defgh	4.7 defg	6.2 abcd	5.7 ab	5.3 abcde	5.1 abc	5.3 abc	5.2 abcd	5.0 abc
F. With steam from nose	7.1 bc	7.2 b	6.9 bcde	5.8 abc	6.7 ab	5.9 bcdef	4.0 defg	4.6 efghi	4.1 g	4.4 ghij	4.3 efgh	5.1 efgh	4.7 defg	5.2 abcde	4.4 bcde	5.1 abcde	4.3 ef	4.7 abc
F. With tongue	2.8 i	3.3 ij	3.3 kl	3.4 hij	3.5 hi	2.8 m	3.9 defg	3.4 jk	4.1 g	4.3 ghij	3.1 jkl	4.1 hijk	4.7 defg	4.6 defghi	4.6 bc	4.7 bcdef	4.6 cdef	4.5 bc
Flexed biceps	4.0 h	4.0 hi	3.7 jk	4.2 fgh	4.1 gh	4.4 ijk	3.5 efg	4.1 ghij	4.1 g	4.8 defgh	3.4 hijkl	4.9 fghi	4.0 g	4.0 hi	3.2 f	4.2 ef	3.2 h	4.7 abc
Flushed f.	5.7 def	5.6 efg	6.0 efg	5.8 abc	6.0 bcde	5.2 efghi	4.5 cd	4.7 efgh	5.1 def	4.8 defgh	4.4 efg	5.2 defg	5.5 abcd	5.7 a	5.1 abc	5.6 a	5.7 ab	5.2 abc
Nauseated f.	7.4 bc	6.5 bcd	7.1 bcd	6.3 a	6.8 ab	5.8 cdef	4.7 bcd	5.3 de	6.1 bc	5.4 cdef	4.9 bcde	6.3 abc	5.7 abc	5.6 ab	5.2 abc	5.2 abcd	6.0 a	5.2 ab
Oncoming fist	4.9 fgh	4.9 gh	4.4 ij	4.5 fg	4.8 fg	4.7 hij	4.1 cdefg	4.3 ghi	4.4 efg	4.7 defgh	4.0 fghij	4.7 fghijk	4.1 g	4.2 ghi	3.7 def	4.4 def	3.3 gh	4.7 abc
Party popper	2.2 i	2.6 jk	2.7 l	3.0 ij	2.9 i	2.9 m	3.3 g	2.5 l	3.8 gh	4.3 hij	2.8 l	3.8 jk	4.4 efg	4.2 ghi	4.4 bcde	3.9 f	4.3 ef	4.6 bc
Persevering f.	7.2 bc	6.9 bc	7.2 bc	6.2 ab	7.2 a	6.1 bcd	4.6 bcd	5.2 ef	5.6 cd	5.2 defg	5.7 bc	6.4 abc	5.8 a	5.7 a	5.2 ab	5.3 abc	6.0 a	5.2 ab
Person in lotus position	3.1 i	3.4 ij	3.1 kl	2.9 j	3.4 hi	5.0 ghi	7.1 a	7.4 a	5.5 cd	5.5 cd	5.4 bcd	6.0 bcde	5.1 abcdef	4.8 bcdefgh	3.4 f	4.8 abcde	5.5 abc	5.5 a
Person shrugging	5.6 ef	5.5 efg	5.8 fgh	5.4 abcde	5.8 cde	5.6 defg	5.1 bc	5.2 ef	5.3 cde	5.4 cde	4.8 cdef	5.6 cdef	5.6 abcd	5.5 ab	4.9 abc	5.2 abcd	5.8 ab	5.1 abc
Pouting f.	8.3 a	8.3 a	8.1 a	6.0 ab	7.4 a	6.0 bcde	3.3 fg	4.3 ghi	3.9 gh	4.3 hij	3.9 ghijk	5.3 def	4.2 fg	4.9 abcdefg	4.5 bc	4.9 abcde	3.8 fgh	4.8 abc
Sleeping f.	4.9 fgh	4.9 gh	5.0 hi	4.6 efg	5.2 ef	6.7 b	7.1 a	6.9 abc	7.1 a	6.7 ab	8.1 a	7.0 a	5.5 abcd	5.5 abc	5.5 a	5.1 abcd	5.9 ab	5.2 ab

Smiling f. With	2.4	2.7	2.7	3.1	3.6	3.0	4.8	4.4	4.3	4.6	3.4	4.3	4.1	3.8	3.7	3.9	3.8	4.4
sunglasses	i	jk	l	ij	hi	lm	bcd	fghi	fg	efghi	hijkl	ghijk	g	i	ef	f	fgh	bc
Warning	6.0	6.1	5.7	5.4	6.0	5.4	4.2	4.6	4.3	4.5	3.8	4.9	4.8	4.7	4.4	3.9	4.0	5.4
	de	cde	fgh	bcde	bcde	defgh	cdef	efghi	fg	ghij	ghijk	fghi	cdefg	cdefghi	bcde	f	fgh	a
Yawning f.	6.1	5.7	6.6	6.0	6.0	7.7	6.5	6.4	7.3	7.1	8.0	7.0	5.5	5.4	5.5	5.5	5.9	5.2
	de	defg	bcdef	ab	bcde	a	a	bc	a	a	a	a	abcd	abcd	a	ab	ab	ab
Zzz	5.0	4.9	5.1	5.0	5.3	6.5	7.3	7.1	6.9	6.5	8.0	7.0	5.4	5.4	5.5	5.3	6.0	5.0
	fg	gh	ghi	cdef	ef	bc	a	ab	ab	ab	a	ab	abcd	abc	a	abc	a	abc

Notes. *) Tukey's HSD used for post-hoc tests. Within columns, emoji that share a letter are not significantly different at the 5% level.

Part 5a of Supplementary Material

Means for the 18 PAD variables (all face emoji in orange, all non-face emoji in green) on the 24 emoji in Study 1 (NZ). PAD variables are labelled: P1 to P6 for *Pleasure* (P), A1 to A6 for *Arousal* (A) and D1 to D6 for *Dominance.* Table 1 has full details, but generally: P - low anchor (1) is associated with pleasure and high anchor (9) is associated with displeasure; A - low anchor (1) is associated with arousal and high anchor (9) is associated with non-arousal; D - low anchor (1) is associated with dominance and high anchor (9) is associated with submissiveness.

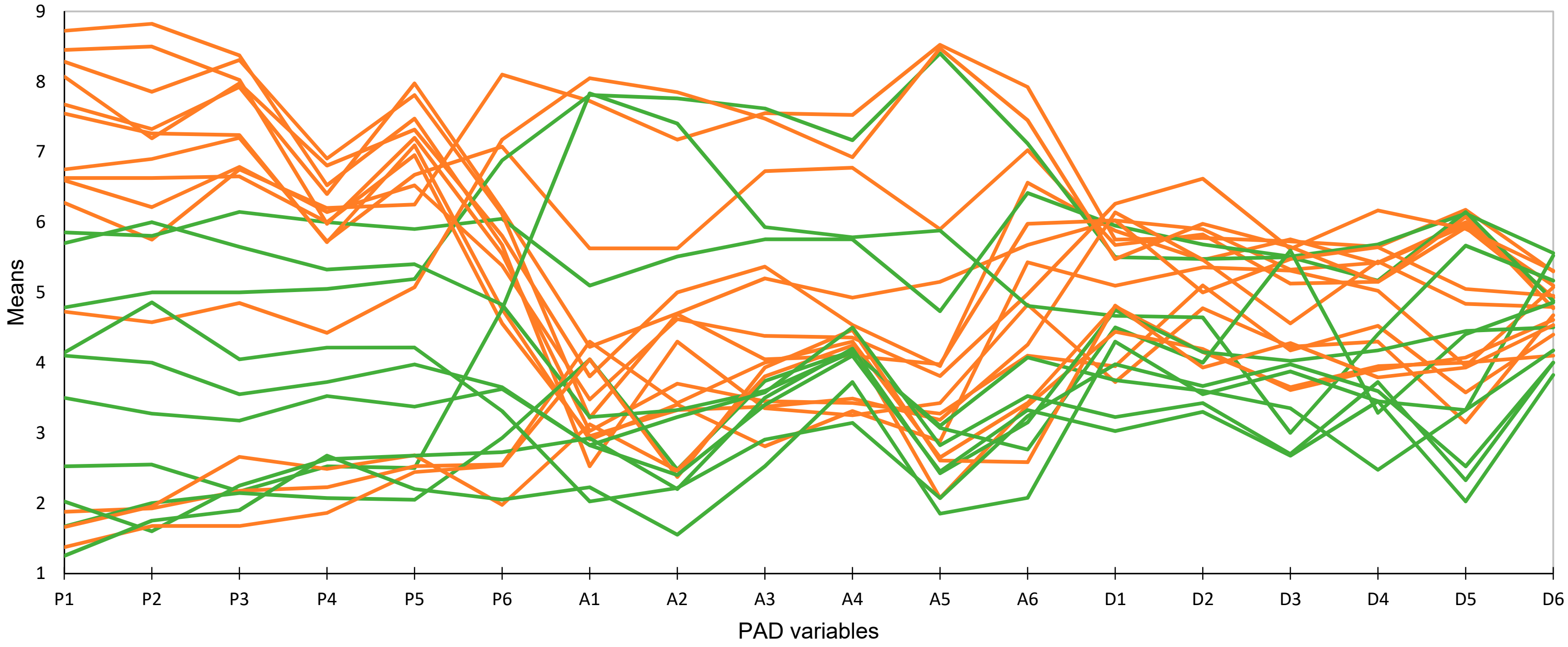

Part 5b of Supplementary Material

Means for the 18 PAD variables (all face emoji in orange, all non-face emoji in green) on the 24 emoji in Study 2 (UK). PAD variables are labelled: P1 to P6 for *Pleasure* (P), A1 to A6 for *Arousal* (A) and D1 to D6 for *Dominance.* Table 1 has full details, but generally: P - low anchor (1) is associated with pleasure and high anchor (9) is associated with displeasure; A - low anchor (1) is associated with arousal and high anchor (9) is associated with non-arousal; D - low anchor (1) is associated with dominance and high anchor (9) is associated with submissiveness.

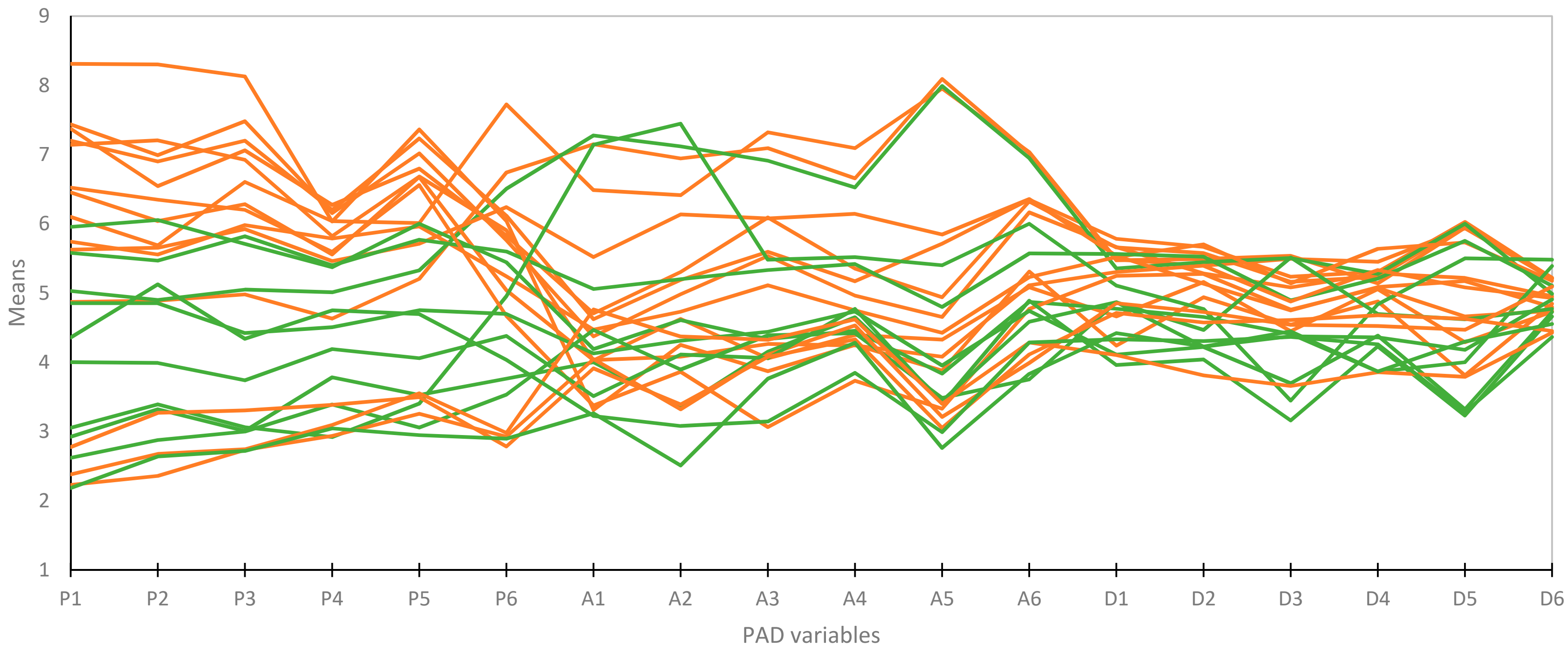

Part 6a of Supplementary Material

Averages* for the 3 PAD dimensions in Study 1 (NZ). For P dimension (*Pleasure*), the low anchor (1) is associated with pleasure and the high anchor (9) is associated with displeasure; for A dimension (*Arousal*), the low anchor (1) is associated with arousal and the high anchor (9) is associated with non-arousal; for D dimension (*Dominance*), the low anchor (1) is associated with dominance and the high anchor (9) is associated with submissiveness. Table 1 in the main text has the full set of scale anchors.

Emoji name	Pleasure	Arousal	Dominance
Beating heart	2.1 j	3.4 efgh	4.4 cdef
Clapping hands	2.3 j	3.1 ghi	4.3 def
Collision	4.1 gh	2.6 hi	3.6 fgh
Exploding head	6.6 abc	3.5 efgh	5.0 abcde
Expressionless face	6.7 abc	6.3 b	5.4 abc
Face savoring food	1.9 j	3.3 fghi	4.2 efg
Face screaming in fear	6.2 bcde	3.4 efgh	5.2 abcd
Face vomiting	7.5 a	4.4 de	5.3 abc
Face with steam from nose	7.3 ab	3.5 efgh	4.3 def
Face with tongue	2.3 j	3.2 ghi	4.2 efg
Flexed biceps	3.4 hi	3.4 efgh	3.2 gh
Flushed face	6.2 bcde	4.3 def	5.9 a
Nauseated face	7.2 ab	4.9 cd	5.6 a
Oncoming fist	3.8 hi	3.1 ghi	3.1 h
Party popper	2.0 j	2.3 i	3.8 fgh
Persevering face	7.2 ab	5.0 cd	5.6 a
Person in lotus position	2.8 ij	6.2 b	4.6 bcdef
Person shrugging	5.9 cdef	5.5 bc	5.7 a
Pouting face	7.6 a	3.6 efgh	4.1 efg
Sleeping face	5.1 fg	7.7 a	5.5 ab
Smiling face with sunglasses	2.2 j	3.7 efg	4.1 efg
Warning	5.5 def	3.6 efgh	3.7 fgh
Yawning face	6.6 abcd	7.7 a	5.7 a
Zzz	5.3 ef	7.6 a	5.4 ab

Notes. *) Tukey's HSD used for post-hoc tests. Within columns, emoji that share a letter are not significantly different at the 5% level.

Part 6b of Supplementary Material

Averages* for the 3 PAD dimensions in Study 2 (UK). For P dimension (*Pleasure*), the low anchor (1) is associated with pleasure and the high anchor (9) is associated with displeasure; for A dimension (*Arousal*), the low anchor (1) is associated with arousal and the high anchor (9) is associated with non-arousal; for D dimension (*Dominance*), the low anchor (1) is associated with dominance and the high anchor (9) is associated with submissiveness. Table 1 in the main text has the full set of scale anchors.

Emoji name	Pleasure	Arousal	Dominance
Beating heart	3.1 kl	4.1 ghi	4.8 cdefgh
Clapping hands	3.4 jkl	4 ghij	4.5 ghijk
Collision	4.6 hi	3.4 ij	4.2 ijkl
Exploding head	6 cde	3.7 hij	5.0 bcdefg
Expressionless face	5.8 def	6 bc	5.1 abcdef
Face savoring food	2.7 l	3.9 ghij	4.7 defghi
Face screaming in fear	6 cde	4.3 fgh	5.2 abcde
Face vomiting	6.8 ab	5.1 de	5.2 abcd
Face with steam from nose	6.6 bc	4.4 fg	4.7 defgh
Face with tongue	3.2 kl	3.8 ghij	4.6 efghij
Flexed biceps	4.1 ij	4.1 gh	3.9 l
Flushed face	5.7 def	4.8 ef	5.5 ab
Nauseated face	6.6 bc	5.5 cd	5.5 ab
Oncoming fist	4.7 ghi	4.4 fg	4.1 jkl
Party popper	2.7 l	3.4 j	4.3 hijkl
Persevering face	6.8 ab	5.4 cd	5.5 a
Person in lotus position	3.5 jk	6.2 b	4.9 cdefg
Person shrugging	5.6 ef	5.2 de	5.3 abc
Pouting face	7.4 a	4.1 gh	4.5 fghij
Sleeping face	5.2 fgh	7.2 a	5.5 ab
Smiling face with sunglasses	2.9 kl	4.3 fgh	3.9 kl
Warning	5.8 def	4.4 fg	4.5 fghij
Yawning face	6.4 bcd	7.1 a	5.5 ab
Zzz	5.3 efg	7.1 a	5.4 ab

Notes. *) Tukey's HSD used for post-hoc tests. Within columns, emoji that share a letter are not significantly different at the 5% level.

Part 7a of Supplementary Material

Two-dimensional spaces following Principal Components Analysis for PAD ratings of 24 emoji in Study 1 (NZ). A) Variables plot spanned by PC2 and PC3 and B) observations plot spanned by PC2 and PC3. For variables plot, colours are used to visually highlight the PAD dimensions, with blue for P1 to P6, green for A1 to A6 and red for D1 to D6.

A)

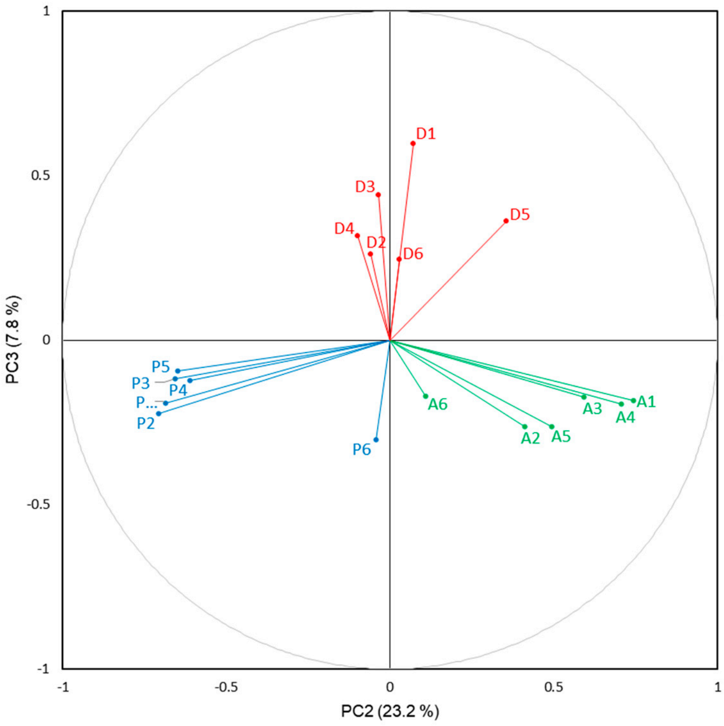

B)

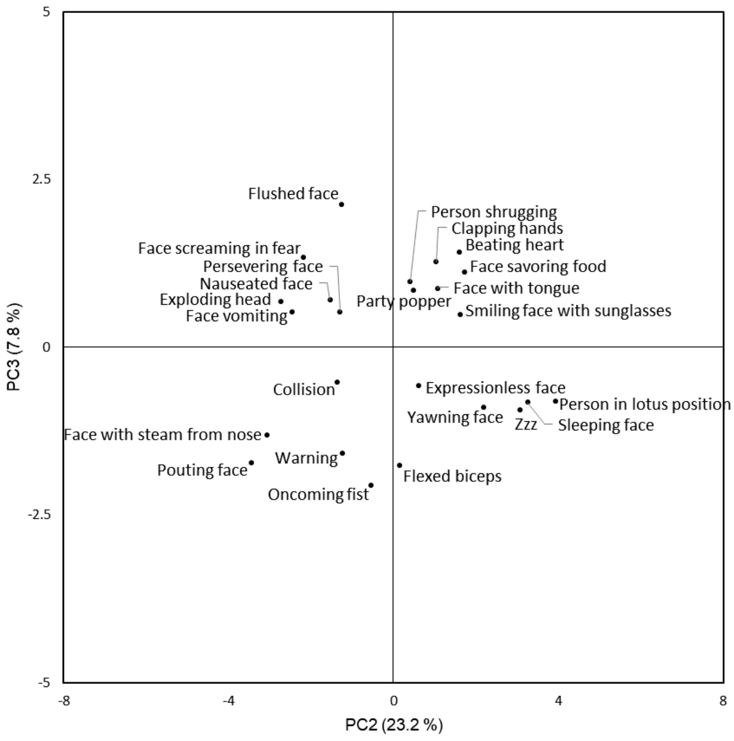

Part 7b of Supplementary Material

Two-dimensional spaces following Principal Components Analysis for PAD ratings of 24 emoji in Study 2 (UK). A) Variables plot spanned by PC2 and PC3 and B) observations plot spanned by PC2 and PC3. For variables plot, colours are used to visually highlight the PAD dimensions, with blue for P1 to P6, green for A1 to A6 and red for D1 to D6.

A)

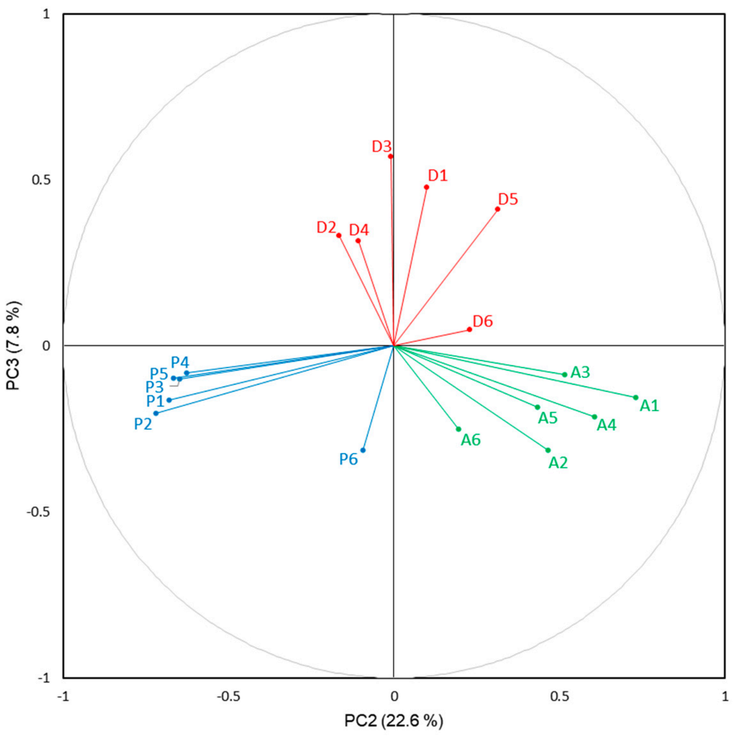

B)

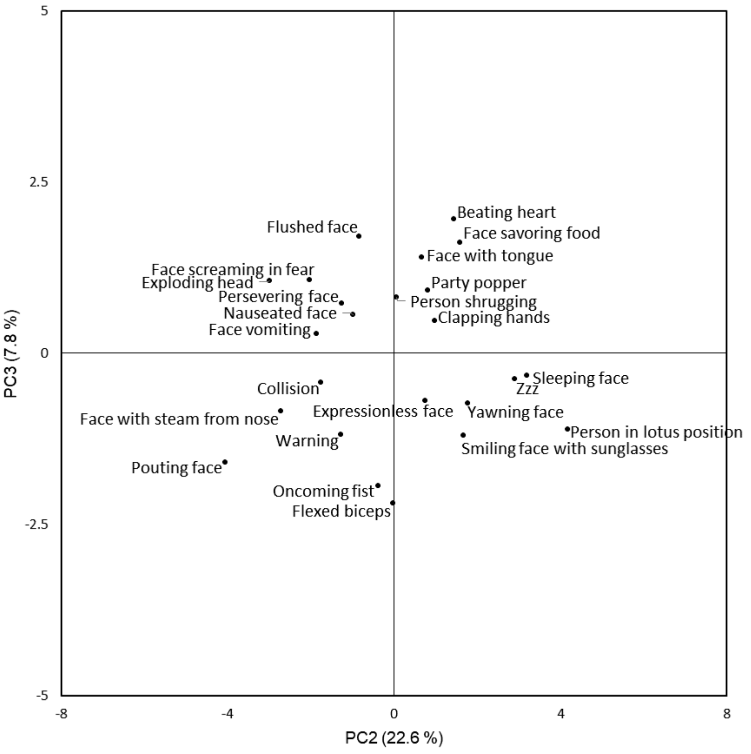

Part 8 of Supplementary Material

Profile plot following hierarchical cluster analysis (Euclidian distance, Ward's method) on merged data from Study 1 and Study 2 that were entered as mean centred (within participants). Two retained clusters of roughly even size (48% in Cluster 1 and 52% in Cluster 2), where participants were roughly evenly distributed between studies (Study 2 participants allocated with 46% in Cluster 1 and 54% in Cluster 2).

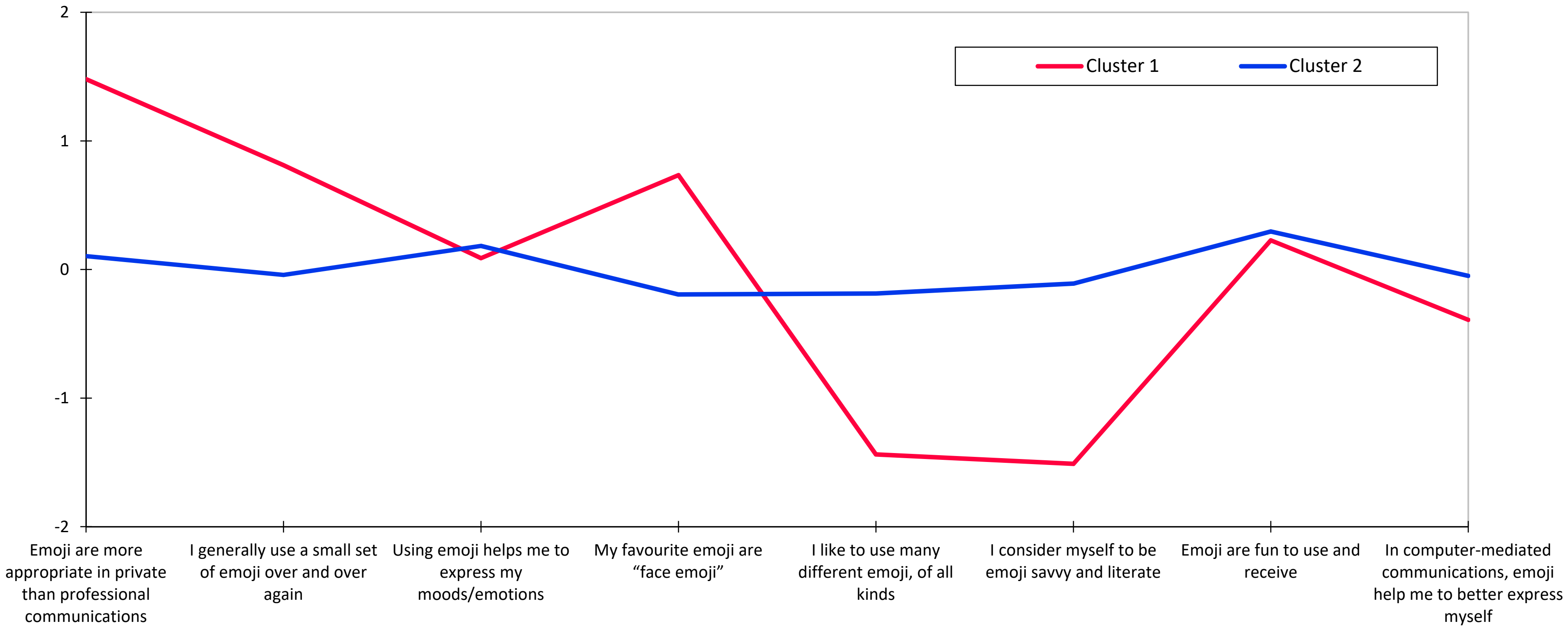

Supplement: Supplementary file 1 [file foods-10-02880-s001.zip › foods-1460092-supplementary.pdf]
